# Needle penetration test - qualifying examination of 3D printable silicones for vascular models in surgical practice

**DOI:** 10.1186/s41205-021-00110-y

**Published:** 2021-08-13

**Authors:** Thore von Steuben, Christoph Salewski, Alexander B. Xepapadeas, Moritz Mutschler, Sebastian Spintzyk

**Affiliations:** 1 Section Medical Materials Science and Technology at the Department of Thoracic and Cardiovascular Surgery, University Hospital Tübingen, Osianderstr, 2-8, 72076 Tübingen, Germany; 2 Department of Thoracic and Cardiovascular Surgery, University Hospital Tübingen, Tübingen, Germany; 3 Department of Orthodontics, University Hospital Tübingen, Tübingen, Germany; 4 Department of Prosthodontics, University Hospital Tübingen, Tübingen, Germany

**Keywords:** Material testing, Needle penetration test, Rapid prototyping, Additive manufacturing, 3D printable silicones, DIY lab-equipment, Surgical training, Medical training, 3D printing

## Abstract

**Background:**

During cardiogenic shock blood circulation is minimal in the human body and does not suffice to survive. The extracorporeal life support system (ECLS) acts as a miniature heart-lung-machine that can be temporarily implanted over major vessels e.g. at the groin of the patient to bridge cardiogenic shock. To perform this procedure in an emergency, a proper training model is desirable. Therefore, a 3-dimensional-printable (3D) material must be found that mimics large vessel needle penetration properties. A suitable test bench for material comparison is desirable.

**Methods:**

A test setup was built, which simulated the clinically relevant wall tension in specimens. The principle was derived from an existing standardized needle penetration test. After design, the setup was fabricated by means of 3D printing and mounted onto an universal testing machine. For testing the setup, a 3D printable polymer with low Shore A hardness and porcine aorta were used. The evaluation was made by comparing the curves of the penetration force to the standardized test considering the expected differences.

**Results:**

3D printing proved to be suitable for manufacturing the test setup, which finally was able to mimic wall tension as if under blood pressure and penetration angle. The force displacement diagrams showed the expected curves and allowed a conclusion to the mechanical properties of the materials. Although the materials forces deviated between the porcine aorta and the Agilus30 polymer, the graphs showed similar but still characteristic curves.

**Conclusions:**

The test bench provided the expected results and was able to show the differences between the two materials. To improve the setup, limitations has been discussed and changes can be implemented without complications.

**Supplementary Information:**

The online version contains supplementary material available at 10.1186/s41205-021-00110-y.

## Background

Potential applications of printable silicones for surgical practice models are a field of rising interest [1]. The progress in 3-dimensional (3D) imaging enables to generate models based on e. g. computer-tomography- (CT) or magnetic-resonance-imaging-data (MRI), which can be 3D printed in various materials. For example, visualizing vascular systems is feasible for preparation of surgical interventions. Materials with tissue-like mechanical properties form realistic phantoms for surgeons to practice [2]. These models could improve teaching clinical skills and help planning complex interventions.

Such training models are particularly important for teaching rapid intervention procedures in situations where quick and flawless execution is crucial for the patient’s survival. This is the case for the extracorporeal life support system (ECLS), which acts as a miniature heart-lung-machine in patients suffering from cardiogenic shock [3]. The device is temporarily implanted over major vessels such as in the patient’s groin. Therefore, needles must be punctured into the Arteria and Vena femoralis. After stretching the openings in the tissue with special dilatators, cannulas with a large diameter are placed in the vessels. The collected venous blood will then become oxygenated in an oxygenator and returned to the patient via the arterial cannula.

To find a fitting material a test suitable test setup is desirable. We designed a method to mimic the penetration of a large as it is done in the Seldinger-technique [4]. To do so, we simulated blood pressure and insertion angle on a flat circular polymer sample. The simulation then has been combined with a needle penetration test and enabled the comparison of materials in this particular situation. Porcine aorta was chosen for comparison, because of similar anatomy and structure to human tissue [5]. Because a low Shore A hardness is an indicator for the hardness of polymers and we were looking for a soft material, similar to human tissue, we preselected Agilus30 from Stratasys [6] as the 3D printed silicon of choice. The industrial norm ISO 11040-4 (F) describes the method of choice for needle penetration tests [7]. For the proposed test setup, the test protocol was adjusted to fit the needs of this investigation. The tests above were designed to deliver a proof of concept for the new method presented in this work.

We came out with a test bench, which allows the comparison of the mechanical properties of different polymers and organic tissue in case of a needle penetration. For 3D printing is easy accessible, the effort for reproduction and changes to the setup are minimized. Different phases of needle penetration could be withdrawn from the force displacement diagrams. The tests showed the expected results and proofed to be suitable for material comparison.

## Materials and methods

The main test stand was designed to be mounted on a ZwickRoell Z010 universal testing machine [8]. All parts and clamps were designed, prototyped, refined and finally manufactured by 3D printing. Therefore, the reproduction of the method is easily actionable and the method can be enhanced with a minimum of effort.

### 3D printing

The Prusa i3 MK3 was used for printing the final setup [9]. The Formlabs Form 2 was used to produce the guiding platform for the porcine samples [10]. The Settings for the Formlabs Form 2 printer were a layer thickness of 0,025 mm and the support structures automatically generated onto the bottom side of the part. For the Prusa i3 MK3 printed parts we used the “0.15mm QUALITY MK3” pre-set. Support structures were also automatically generated.

### Agilus30

The Agilus30 is a 3D printable material that, due to its low Shore A hardness of 30, seemed suitable for an aortic model. The Shore A hardness of a 2 mm thick aortic tissue is approximately 28 [11]. In addition, first tests were made in the area of vascular modelling and suitability for medical training purposes [6, 12]. The thickness of the samples of 2 mm was chosen based on surgical experience.

### Dissection of the porcine hearts

Because porcine tissue is similar to that of humans in its circular and longitudinal stiffness and easy to obtain, porcine aorta was chosen for comparison [5].

Before the aortae were ready to be further processed, they had to be dissected from the hearts. The trachea was separated first. Then, connective tissue between the aorta and the pulmonary artery was cut apart, so the excess vessels could be removed individually. Finally, the aortae were separated from the heart at the aortic root and clearly identified by looking at the exposed aortic valve. To flatten out the aortic tissue, the obtained ascending aorta with the aortic arch was opened axially in line with the supraaortic arteries. Deviations in thickness naturally occur and were therefore neglected.

### Punching of samples

**Fig. 1:**
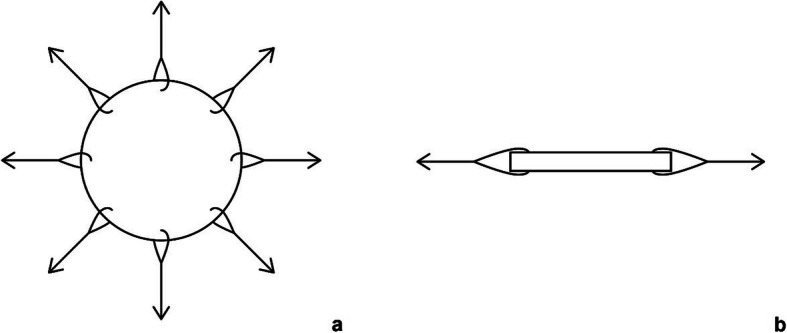
A scetch drawing of a sewn sample from the top (**a**) and as a disection view (**b**)

Samples were punched out of the dissected aorta and the Agilus30 sample mat (see Additional files 1 and 2) using a 20 mm hole punch according to DIN 7200 resulting in samples of the same diameter. The thickness was 2 mm for the Agilus30 samples and approximately 2 mm for those from the porcine aorta [13]. If needed, scissors (see Additional files 1 and 2) were used to detach the sample from the rest of the tissue/material (see Fig. 1).

### Sewing of the porcine samples

The punched samples had to be connected to weights by a suture. With appropriate suture material (see Additional files 1 and 2) a loop was sewn into the sample perpendicular to the surfaces. A cardboard template was used for the placement of the seams, on which the correct position of the stiches was drawn beforehand. The sutures were arranged at 0, 45, 90, 135, 180, 225, 270 and 315 degrees (see Fig. 1).

### Conceptualization

Before the conceptualization began, a list of requirements was set. The list contained forces that had to act on the sample, to simulate a realistic state of tension. First there is the force of the needle hitting the vessel. This required a counter bearing, which arises physiologically by the internal blood pressure. The blood pressure p for calculations was 120 mmHg systolic. The axial and tangential states of tensile stress inside the wall (see Formula 1) are depending on the internal pressure, the internal diameter

$${\sigma}_t=\frac{p\cdot {d}_m}{2\cdot {s}_w};{\sigma}_a=\frac{p\cdot {d}_m}{4\cdot {s}_w}$$

Formula 1 Tangential (σ_t_ [N/mm^2^]) and axial (σ_a_ [N/mm^2^]) wall tension resulting from the variables internal pressure (p [N/mm^2^]), mean diameter of the vessel (d_m_ [mm]) and wall thickness (s_w_ [mm]).

and the wall thickness and were calculated by a formula for pipeline construction from DIN EN 13480 [14]. The mean diameter of the aorta for the calculation was d_m_ 33 mm and the wall thickness s_w_ 2 mm. If a sample is taken from this wall, the wall tensions can be used to calculate the forces that must act on the edges to simulate the physiological internal stress. To get the forces, the circumference area is calculated with the sample’s radius and thickness. Because the forces act on one eighth of the area it is divided by eight (see Formula 2). The samples thickness s_s_ was 2 mm and the sample’s diameter d_s_

$${A}_{\frac{1}{8}}=\frac{d_s\cdot \pi \cdot {s}_s}{8}$$

Formula 2: Circumference area of the sample (${A}_{\frac{1}{8}}$ [mm^2^]) resulting from the variables sample diameter (d_s_ [mm]) and the sample thickness (s_s_ [mm]) devided by eight.

20 mm. The area can now be used to calculate the acting force out of the tensions mentioned above. The axial and tangential tensions are simply divided by their area, while the diagonal tensions are combinations of both. Because the angle is 45°, the tension is both, half axial and halt tangential. In the end it is also divided by the area (see Formula 3,4 and 5). To apply the forces on the samples in a

$${F}_1={\sigma}_a\cdot {A}_{\frac{1}{8}}$$

Formula 3: Force to apply to the sample at the positions 0° and 180° (F_1_ [N]) resulting from the variables axial wall tension (σ_a_ [N/mm^2^]) and one eighth of the circumference area of the sample (${A}_{\frac{1}{8}}$ [mm^2^]).

$${F}_2=\frac{\left({\sigma}_a+{\sigma}_t\right)}{2}\cdot {A}_{\frac{1}{8}}$$

Formula 4: Force to apply to the sample at the positions 45°, 135°, 225° and 315° (F_2_ [N]) resulting from the variables axial wall tension (σ_a_ [N/mm^2^]), tangential wall tension (σ_t_ [N/mm^2^]) and one eighth of the circumference area of the sample (${A}_{\frac{1}{8}}$ [mm^2^]).

$${F}_3={\sigma}_t\cdot {A}_{\frac{1}{8}}$$

Formula 5: Force to apply to the sample at the positions 0° and 180° (F_3_ [N]) resulting from the variables tangential wall tension (σ_t_ [N/mm^2^]) and one eighth of the circumference area of the sample (${A}_{\frac{1}{8}}$ [mm^2^]).

constant manner, we decided to use weights. The weights are applied by threads, which were guided through holes in the sample holder. The upcoming friction was neglected. The masses of the weights were calculated by dividing the forces with the standard acceleration due to gravity (see Formula 6).

$${m}_{1,2,3}=\frac{F_{1,2,3}}{g}$$

Formula 6: Masses (m_1,2,3_ [kg]) to apply on the samples for generating the calculated Forces (F_1,2,3_ [N]). The forces have been divided by the standard acceleration due to gravity (g).

The calculation of the weights has been previously described by C. Salewski et al. [15]. The Formulas enable the examiner to adjust the tension for an individual tissue in its special state of tension.

**Fig. 2:**
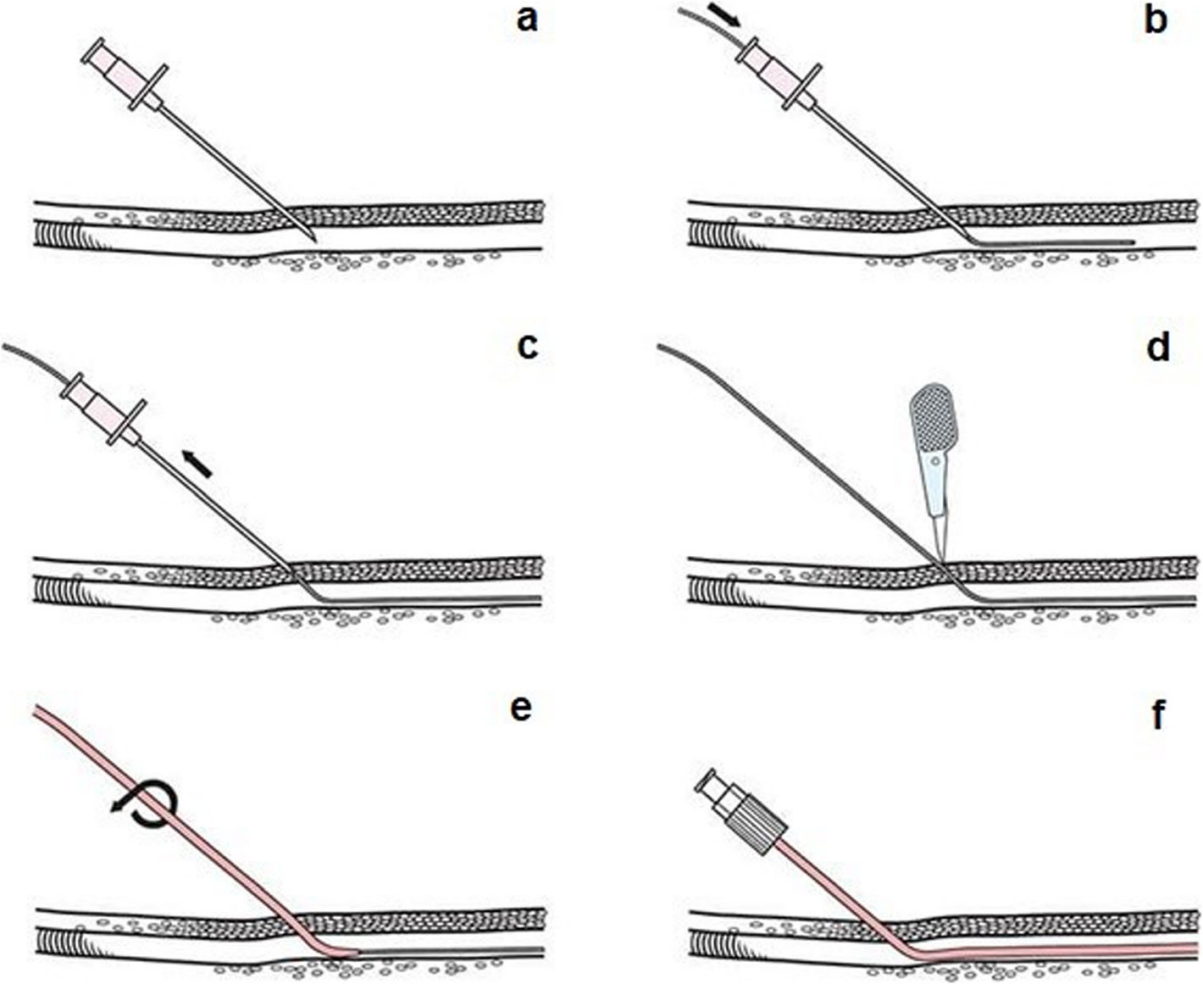
A step by step scheme of the Seldinger technique. The needle puncturing the vessle (**a**). After puncture a wire is going to be inserted into the vessle as guidance fort he cannula or dilatators if needed (**b**). The needle can now be withdrawn from the body (**c**). A insision (**d**) helps the cannula or dilatator (**e**) to get inserted in the vessle with a rotational move. In the end the cannula stays in the patient and is ready to be used (**f**)

Another important part was performing a realistic puncture. Vessels should be punctured at an angle of 45 degrees according to the Seldinger-technique [4, 16] (see Fig. 2). Since the needle in the Zwick Z010 must always be guided vertically, the sample holder needed a 45-degree inclined position. To compensate for possible deviations in the vertical alignment of the test machine, needle or sample holder had to be adjustable in the orthogonal level.

### Final test bench

**Fig. 3:**
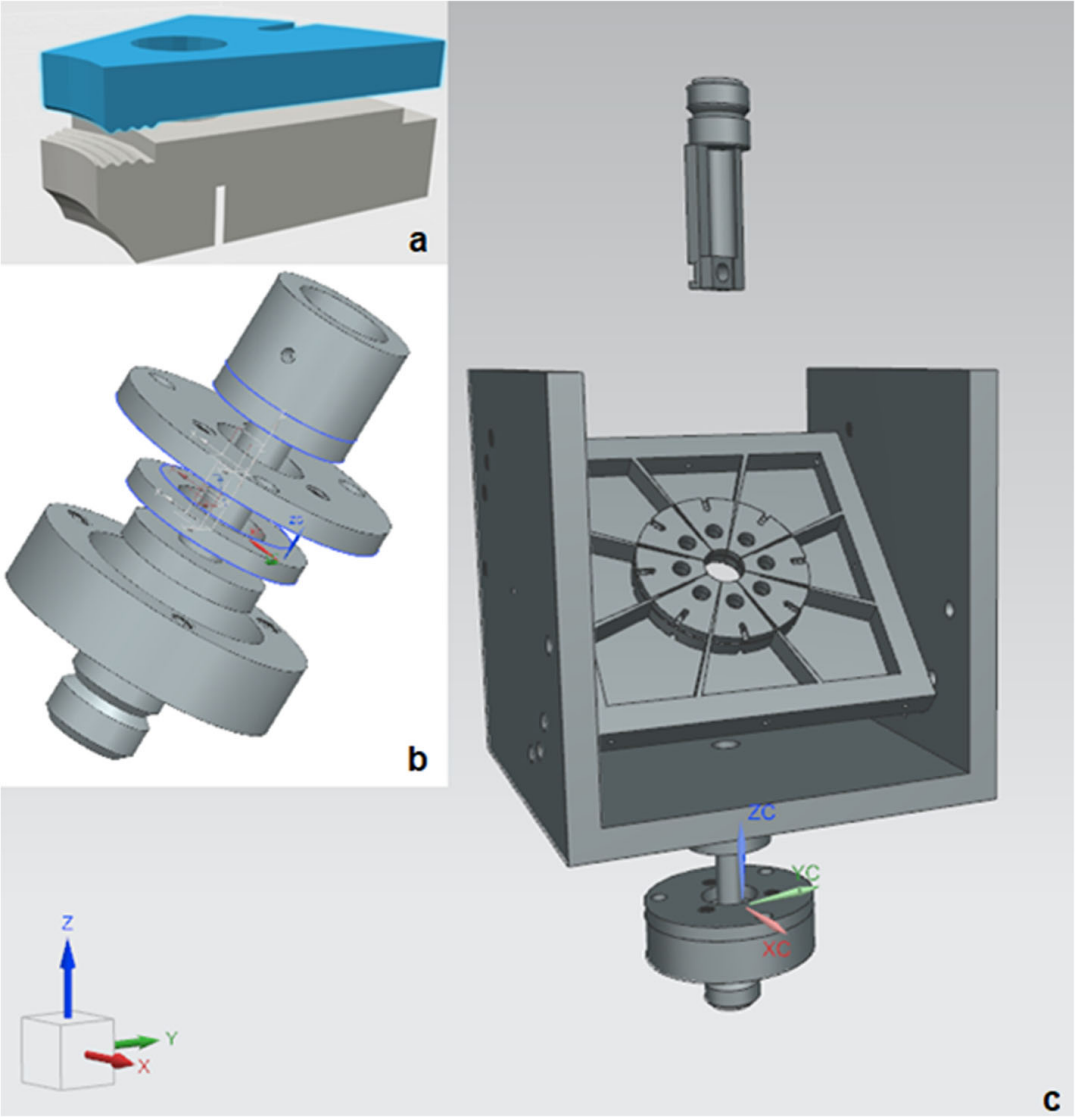
3D-model of the composed printable parts of the final test bench (right). A 3D-model of one sample clamp in the correct arragnement (top left). Explosion model of the horizontal arrangement socket (central left). (See Supplementary Materials)

**Fig. 4:**
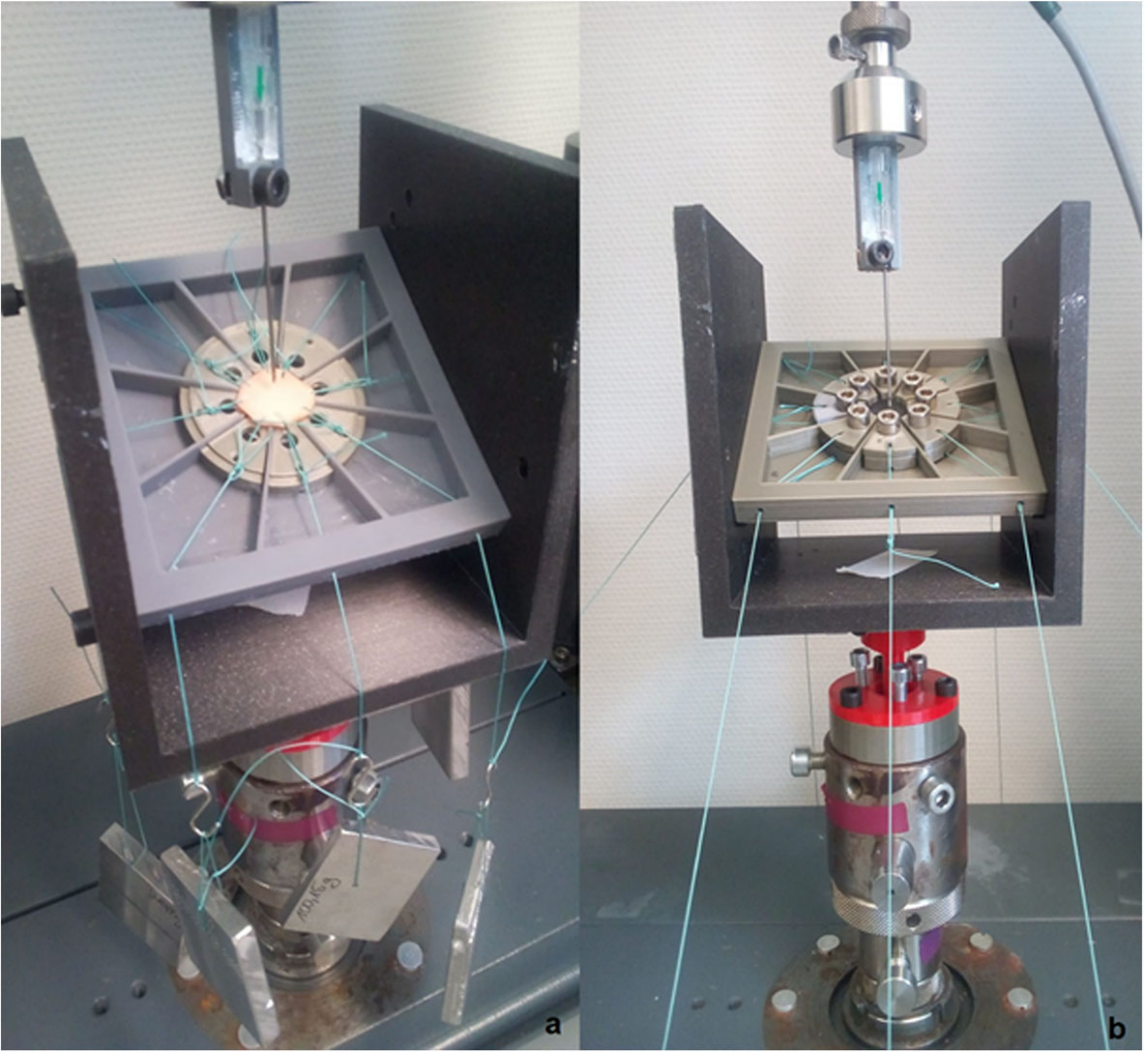
Final test bench in use. On the left the final setup in case of the porcine aorta ca be seen (a). The lower part of the clamps were glued on the surface of the platform as a counter bearing. To the right is the final setup in case of the tests with the Agilu30. The clamps are used to stretch the sample instead of sewing the strings directly in the material as it was done for the aorta. Because of the closed clamps and the high screws the sample unter the needle is bearly visable

A brief description of the test bench has been made by C. Salewski [15]. For the sake of a complete explanation this part contains the description mentioned above and offers additional details, which are essential for reproduction. Additions have been made in terms of the printer and machine settings, explanations of the purpose of design decisions, the overall construction of the clamps and the whole design and use of the adjustment base. The final test bench consisted of five major parts. A sample holder with angle options of 90, 60, 45 and 30 degrees. 45 and 30 degrees were not feasible while testing due to limited possibilities of guiding the needle and narrow space between the needle and the claps. The tilt able platform with its bearings guiding the clamps towards the centre. A socket for alignment adjustments between sample holder and testing machine. The needle holder, which could guide the puncture needle in an aligned manner by clamping it at the middle of the tube. The direction of the needle opening is fixed by a plaster mould on the needle holder. And finally, the weights, made of a bismuth-tin-alloy, which ensured the constant resilience of the sample (see Fig. 3). The predefined weights were hung on the clamps or threads of the sample (see Fig. 4). All polymer components, except for the tilting surface for the aorta, were printed with the Prusa i3 MK3 out of polylactide (PLA) (see Additional files 1 and 2). The tilt surface for the porcine aorta was printed in Formlabs Form 2 with Formlabs Grey V4 Resin (see Additional files 1 and 2).

Since testing the penetration force of a needle through a membrane represents a typical pressure test, this was chosen for the comparison of the material properties [7]. The needle was attached to a movable piston and aligned vertically with the opening pointing towards the lowest point of the tilting surface. The additional base inserted between the tool holder and the sample holder allowed alignment on a plane orthogonal to the needle. Alignment was determined manually by the examiner. The needles were always replaced together with the sample after each punctuation. A 17 gauge (G) needle (see Additional files 1 and 2) was used, as it is also used in clinical practice for the Seldinger-technique. The sample is round with a diameter of 20 mm and a thickness of 2 mm. The setup is also able to test samples of different thickness. The clamps or sutures were arranged at 0, 45, 90, 135, 180, 225, 270 and 315 degrees. It started with the highest position of the angled sample. For the tests, the tilting surface was set to 60 degrees.

### Measurement setup

The test device used was the Zwick Z010 universal testing machine [8]. Despite the maximum test force of 500 N, it can dose low penetration forces and deliver them precisely. With a test speed of 0.001-1800 mm / min in the z direction, the tolerance was high enough in both directions within the required range. Due to the low expected forces, no preload was selected. Break detection was deactivated.

The maximum load was chosen based on medical experience and values expected from the literature [17]. The test speed was adjusted based on puncture force tests already carried out [18].

The parameters used are listed below (see Table 1).

**Table 1:** Zwick Z010 Settings for the test series

Parameter	Value
maximum axial force	100 N
preload	0 N
velocity	1 mm/min
break detection	off

## Results

The test setup was fully functional. The cannula for the penetration was stable aligned by the holder and easy to be changed. The overall alignment of the bench went well and the adjustment base was firmly screwed. Although the top of the adjuster was thin and flexible it remained in a stable condition throughout the tests. The samples, although they were only fixed by threads, remained in place while the whole process and could quickly be changed. The platform change after the Agilus30 tests went without any complication so the rest of the setup remained in the same position throughout both test rows.

**Fig. 5:**
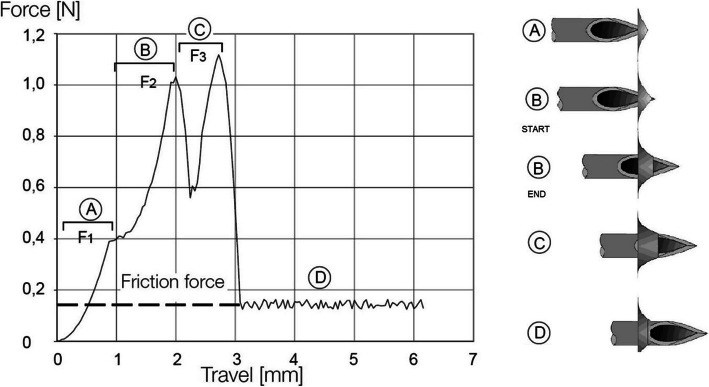
Example force displacement diagramm from ZwickRoell (right) and a visual explaination of the different sections of the diagramm (left). Area A, B and C each describing different force peaks and area D the friction force in the end of a test run

**Fig. 6:**
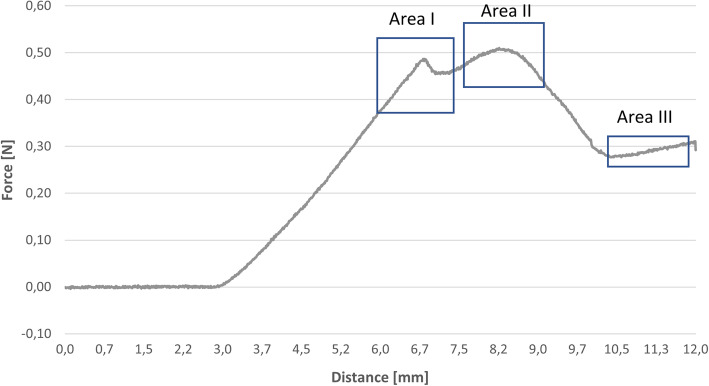
Force displacement diagramm of a porcine aorta sample with description of the three areas of interest. Area I and II marking the two peaks. They describe the maximum elastic and breaking strength of the material. Area III shows a linear section, which refers to the friction force of the needle stuck in the material

The graphs of the porcine aorta (see Fig. 6) differed in some points from the ideal graph (see Fig. 5) for qualitative analysis but basically showed a similar progress as the exemplary diagram [19].

The polymer was evaluated in the same way. In general, the samples showed similar curves with a slight scatter of the maximum values and much steeper trends. Instead of the linear raising trend of Area III, the trend of the Agilus30 is falling.

## Discussion

Subject of our work was the development of a test setup that is suitable for a sufficient comparison of organic blood vessels and synthetic materials. Further, first tests have been made to get an impression of the producible data. By usage of 3D printing in the whole constructional process, the setup should be versatile in terms of tailoring to specific tissues and vessel sizes.

**Fig. 7:**
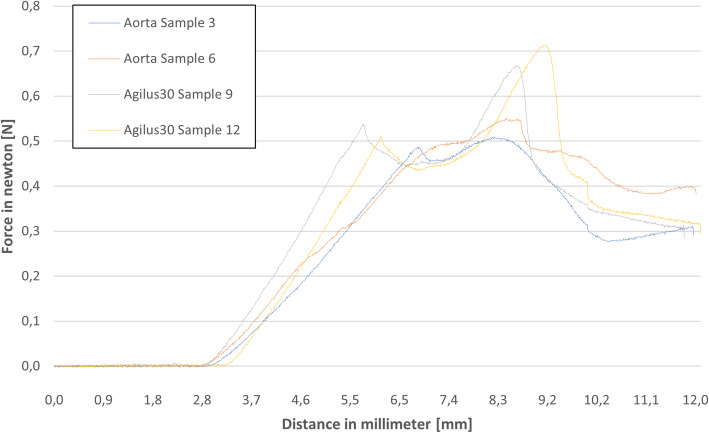
Qualitative comparison of each two samples of porcine aorta (samples 3 and 6) and Agilus30 samples (samples 9 and 12) in form of a force displacement diagram

The obtained force displacement graphs (see Figs. 6 and 7) matched our expectations. Differences can be explained due to the different nature of the test. The first peak corresponds to the first peak in the exemplary graph. Surface tension raises and drops suddenly after its maximum. The last two example peaks were fused because of the thickness of the sample. It rises, due to the increase of the needle diameter at the tip, while it proceeds through the material. The cutting edge is longer than the sample thickness, therefore the cutting area decreases already when the tip exits the sample. At the maximum in Area II the decrease of the cutting area overcomes the increase of diameter, which results in the decrease of force. At last, Area III corresponds to the friction force of the example. We assume that the force increases in the porcine aorta are caused by abrasion of its fluid components, which results in a dryer surface with higher friction. The abrasion of material on the Agilus30 sample causes an increasing diameter of the hole, which results in a decrease of pressure on the needle and therefore a decrease in the frictional force. Due to the lack of measuring the exact thickness of the porcine samples, higher deviations can be explained. For a thorough statistical analysis of material properties additional tests must be performed.

Although the bench delivered sufficient results, a fabrication in aluminium would increase stability and prevent deformation due to the force applied at the screw connections. A perfectly centred test bench would also be of advantage. Manual centring and alignment of the needle should be made independent from the skills of the examiner. To enable steeper angles of the sample holder and make it independent of the alignment, minimal constructional changes on the clamps are needed. Clamps, which support both synthetic and organic material are desirable. It would increase preparation time of organic samples and comparability with synthetic materials.

The expected curves were congruent with reality. The test bench seems to be suitable for determining material properties in complex stress combinations during needle penetration. With the current setup, samples can be changed within 10–15 min, which allows for a larger test series in a reasonable amount of time.

In addition, weights and therefor the internal stress can be tailored to almost any vessel in the human body. Bearing in mind, that samples with a thickness of 1 mm or less are not recommended, because the frictional forces of the threads become more relevant. Also, the clamps must be modified to enable optimal holding pressure on the sample. Changes are due to the nature of 3D printing easy and fast to implement into the build, so a tailored setup can be built in no time.

## Conclusions

A test bench was developed and manufactured to enable a comparison between porcine tissue and synthetic materials. It was constructed using 3D printing. Because of this, the effort of reproduction and adaptation is minimal. Rapid prototyping helped with the conceptualization and rapid tooling with the production of the final version. The resulting test bench can successfully simulate a puncture at different angles and displays the data in the form of a force-displacement diagram. It also simulates the prevailing wall stress states of an aorta by transferring these through calculated forces to the sample. Furthermore, the formulas can be used to tailor the settings on vessel size. Meaning, that there is no need to manufacture a vessel or apply internal pressure for test purposes. The data collected was sufficiently precise and made it possible to compare the two materials. Although slight differences could be determined, important areas of the graphs were similar to an established process.

## Supplementary Information

**Additional file 1.** List of materials.

**Additional file 2.** 3D models.

## Data Availability

The datasets used and/or analysed during the current study are available from the corresponding author on reasonable request.

## Abbreviations

ECLS: Extra corporal life support
3D: 3-dimensional
CT: Computer tomography
MRI: Magnetic resonance imaging
G: Gauge
