# Supplementary material for: Needle penetration test - qualifying examination of 3D printable silicones for vascular models in surgical practice
Source: 3D Print Med. 2021 Aug 13;7:21. doi: 10.1186/s41205-021-00110-y (PMC8362236; doi:10.1186/s41205-021-00110-y)
Supplement: Supplementary file 1 — Additional file 1. List of materials. [file 41205_2021_110_MOESM1_ESM.docx]

| Manufacturer | Description |
| --- | --- |
| Schlachthof Rottenburg | Porcine hearts |
| dent-e-con e.K. | Samplemat Agilus30 120x100x2mm |
| Gedore | 570020 Arc punch 20mm |
| SC-Normteile | Hexagon socket head cap screws – M6x10 - DIN 912  (ISO 4762) – Full thread – stainless steel A2 V2A –SC912 |
| KTH | Hexagon nuts DIN 439 – A2 – steel – V2A – M6 |
| DIZA | Socket head screws M6x14 DIN 912 – strength 8.8 – Hexagon socket |
| - | Bismut-Zinn-Alloy |
| Prusa | Prusament PLA (different colours) |
| - | Gypsum Typ 3 |
| Mersilene | EH7350 75cm Suture |
| Braun | Single use scalpels BA215 |
| Braun | Pean clip |
| Braun | Metzenbaum scissors |
| VYGON | Special cannula REF 219.14 1,4 x 70mm |
| Emil Lux GmbH & Co KG | V-160718 M6x14mm with nuts |
| MAQUET | Catheters & Cannulae Percutaneous Insertion Kit G-137 |
